# Hereditary Syphilis and Appendicitis

**Published:** 1917-09

**Authors:** 


**Hereditary Syphilis and Appendicitis.** Gaucher, Le Prog. Med., Apr. 7, page 118, reports a syphilitic father and mother. There were 3 abortions, 8 births at term, 3 infants dying early. All the surviving children had appendicitis and 3 died.

